# The Biosafety Research Road Map: The Search for Evidence to Support Practices in the Laboratory—*Shigella* spp.

**DOI:** 10.1089/apb.2022.0046

**Published:** 2023-06-05

**Authors:** Stuart D. Blacksell, Sandhya Dhawan, Marina Kusumoto, Kim Khanh Le, Ben J. Davis, Kathrin Summermatter, Joseph O'Keefe, Joseph Kozlovac, Salama Suhail Almuhairi, Indrawati Sendow, Christina M. Scheel, Anthony Ahumibe, Zibusiso M. Masuku, Allan M. Bennett, Kazunobu Kojima, David R. Harper, Keith Hamilton

**Affiliations:** ^1^ Mahidol-Oxford Tropical Research Medicine Unit, Faculty of Tropical Medicine, Mahidol University, Bangkok, Thailand.; ^2^ Centre for Tropical Medicine and Global Health, Nuffield Department of Medicine, Nuffield Department of Medicine Research Building, University of Oxford, Oxford, United Kingdom.; ^3^ Institute for Infectious Diseases, University of Bern, Bern, Switzerland.; ^4^ Ministry for Primary Industries, Wellington, New Zealand.; ^5^ U.S. Department of Agriculture, Agricultural Research Service, Beltsville, Maryland, USA.; ^6^ Abu Dhabi Agriculture and Food Safety Authority, Abu Dhabi, United Arab Emirates.; ^7^ Research Center for Veterinary Science, National Research and Innovation Agency, Indonesia.; ^8^ WHO Collaborating Center for Biosafety and Biosecurity, Office of the Associate Director for Laboratory Science, Center for Global Health, Centers for Disease Control and Prevention, Atlanta, Georgia, USA.; ^9^ Nigeria Centre for Disease Control and Prevention, Abuja, Nigeria.; ^10^ National Institute for Communicable Diseases of the National Health Laboratory Services, Sandringham, South Africa.; ^11^ UK Health Security Agency, Porton Down, United Kingdom.; ^12^ Department of Epidemic and Pandemic Preparedness and Prevention, World Health Organization, Geneva, Switzerland.; ^13^ The Royal Institute of International Affairs, London, United Kingdom.; ^14^ World Organisation for Animal Health (OIE), Paris, France.

**Keywords:** *Shigella* spp., pathogen characteristics, biosafety evidence, knowledge gap analysis, disinfection, inactivation

## Abstract

**Introduction:**

*Shigella* bacteria cause shigellosis, a gastrointestinal infection most often acquired from contaminated food or water.

**Methods:**

In this review, the general characteristics of *Shigella* bacteria are described, cases of laboratory-acquired infections (LAIs) are discussed, and evidence gaps in current biosafety practices are identified.

**Results:**

LAIs are undoubtedly under-reported. Owing to the low infectious dose, rigorous biosafety level 2 practices are required to prevent LAIs resulting from sample manipulation or contact with infected surfaces.

**Conclusions:**

It is recommended that, before laboratory work with *Shigella*, an evidence-based risk assessment be conducted. Particular emphasis should be placed on personal protective equipment, handwashing, and containment practices for procedures that generate aerosols or droplets.

## Introduction

The World Organization for Animal Health, World Health Organization (WHO), and Chatham House are collaborating to improve the sustainable implementation of laboratory biological risk management, particularly in low-resource settings. The Biosafety Research Roadmap project aims to support the application of laboratory biological risk management and improve laboratory sustainability by providing an evidence base for biosafety measures (including engineering controls) and evidence-based biosafety options for low-resource settings. This will inform strategic decisions on global health security and investments in laboratory systems.

This study involves assessing the current evidence base required for implementing laboratory biological risk management, aiming to provide better access to evidence, identifying research and capability gaps that need to be addressed, and providing recommendations on how an evidence-based approach can support biosafety and biosecurity in low-resource settings.

This review describes the general characteristics of *Shigella* spp., and the current biosafety evidence and available information regarding laboratory-acquired infections (LAIs) and laboratory releases are detailed.

## Materials and Methods

A 15 member technical working group (TWG) was formed to develop a Biosafety Research Roadmap (BRM) with the goal of supporting the application of laboratory biological risk management and improving laboratory sustainability by providing an evidence base for biosafety measures.

The TWG conducted a gap analysis for a selected list of priority pathogens on procedures related to diagnostic testing and associated research for those pathogens, including but not limited to sample processing, testing, animal models, tissue processing, necropsy, culture, storage, waste disposal and decontamination. To achieve this, the TWG screened databases, websites, publications, reviews, articles, and reference libraries for relevant data. The main research domains used to perform the literature searches were the ABSA database, Belgian Biosafety Server, CDC reports, WHO reports, PubMed, and internet searches for terms related to biosafety matters, including, for example, inactivation, decontamination, laboratory-acquired infections, laboratory releases and modes of transmission. The summary of evidence and potential gaps in biosafety was divided into five main sections: route of inoculation/modes of transmission, infectious dose, laboratory-acquired infections, containment releases, and disinfection and decontamination strategies.

## General Characteristics

*Shigella* spp. are common human enteric bacterial pathogens belonging to the family Enterobacteriaceae. They are gram-negative nonsporulating rod-shaped facultatively anaerobic bacteria. *Shigella* spp. are classified as Risk Group 2 pathogens^1^ and are transmitted by the fecal–oral route. Communities lacking public health infrastructure to provide safe drinking water are at the most significant risk for large outbreaks; however, outbreaks related to improperly prepared foods are a global problem. The bacteria demonstrate acid-resistant characteristics, and survival is continued through the digestive system.^2^

### Disease Treatment and Prophylaxis

Shigella infection in young children, the elderly, or the immunocompromised may lead to life-threatening diarrheal disease. The *Shigella flexneri* species causes bacterial dysentery. Treatment of severe *Shigella* spp. related infections include rehydration therapy, and antibiotic therapy using ciprofloxacin and azithromycin,^3,4^ although antimicrobial resistance is a significant issue.^5^ In contrast, a *Shigella* spp. infection in healthy well-nourished individuals is usually self-limiting, and antibiotic treatment is not required. However, after symptoms subside, *Shigella* spp. may be shed in the feces for a month.^6^

### Diagnostics

Laboratory procedures commonly used for diagnosing shigellosis are stool sample examination, stool culture, blood culture, polymerase chain reaction, and enzyme-linked immunosorbent assays.^5^ Serological testing of isolated bacteria with antibodies is used to identify the *Shigella* spp.

## Biosafety Evidence

### Modes of Transmission

Shigellosis is transmitted through the fecal–oral route. Infections are most common in developing country locations where sanitation and hygiene are substandard.^7^ Public health measures are urgently needed to provide uncontaminated water for food preparation and handwashing. *Shigella* is highly transmissible between persons during outbreaks and commonly circulates or clusters in high-risk settings such as day-care centers^8^ and men who have sex with men.^9^ Transmission occurs through contaminated food, water, and fomites with infectious fecal matter.^10^

### Infectious Dose

The high frequency of initial and secondary transmission is due to the very low infectious dose of *Shigella* spp. between 10 and 200 organisms^10^ to 10 and 100 organisms.^11^

### Laboratory-Acquired Infections

Shigellosis is one of the most common LAIs. The frequency is likely underestimated due to (1) the self-limiting nature of the infection and (2) the difficulty in pinpointing the source of infection. Although many shigellosis cases in laboratory workers were reported before 1990, fewer cases with incident descriptions and causes can be found in the literature. The most detailed descriptions come from reports of laboratory infections acquired in British clinical laboratories throughout the 1980s.^12–17^ A review of these LAIs shows that most of these occurred while performing routine work, primarily during the manipulation of cultures and secondarily during the manipulation of infected human (serum and stool) specimens.

Inexperience and carelessness were cited as the number one cause of such accidents. It is important to note that manipulations with *Shigella* spp. and other enteric pathogens were performed on the open bench without gloves. Owing to the low infectious dose, trace contamination of the hands may lead to oral inoculation. Since the 1994 study of Walker and Campbell,^18^ reported cases of shigellosis LAIs have declined.

Updated evidence-based biosafety guidance for handling *Shigella* and other enteric pathogens now includes full personal protective equipment (PPE) (i.e., laboratory coat, gloves, and eye protection) and a primary barrier (biosafety cabinet or bench shield) to prevent accidental contamination.^19^ Handwashing with boiled or disinfected water after glove removal is essential. These factors have likely resulted in decreased shigellosis LAIs over the past decades.

### Decontamination and Inactivation

#### Chemical

It is reported that *Shigella* spp. are susceptible to 1% sodium hypochlorite, 70% ethanol, 2% glutaraldehyde, iodine, phenolics, and formaldehyde; however, only a generic reference^19,20^ has been provided, and there is no clear evidence for the effectiveness of these disinfectants although empirical evidence would suggest that these disinfectants are effective. The WHO provides additional recommendations, with practical advice on preparing appropriate concentrations based on the amount of organic material.^21^

#### Thermal and autoclaving

Organisms can be heat killed by steaming using an autoclave for 1 h at 100°C under normal atmospheric pressure.^22^ The autoclave sterilization cycle is very effective, but the time required for steam penetration in a particular load should be validated.

#### Other methods

Sonophotocatalytic disinfection has been reported effective^23^ and 16.5 ppm ozone.^24^

A complete list of the evidence is provided in Table 1.

**Table 1.** Detailed Pathogen Biosafety Evidence for *Shigella* spp.

Overview of the evidence and potential gaps in biosafety				
Method	Details	Evidence (direct quote where available)	References	Evidence gap? (yes/no)
Route of inoculation	Fecal–oral route	“*Shigella* bacteria multiply within colonic epithelial cells, cause cell death and spread laterally to infect and kill adjacent epithelial cells, causing mucosal ulceration, inflammation and bleeding. Transmission usually occurs via contaminated food and water or through person-to-person contact”	^26^	No
		*“Shigella* infection can occur by the fecal–oral route of transmission, person-to-person contact or ingestion of contaminated food or water”	^27^	
Infectious dose	Fecal–oral route Between 10 and 200 pathogenic organisms	“The number of organisms required to cause the disease is usually 10 to 200 due to the low sensitivity to stomach acid and downregulation of antibacterial proteins of the host by the organism”	^10^	No
		“A low infective dose, on the order of 10 to 100 organisms is sufficient to produce disease. It is typically transmitted by contaminated food and water or by direct contact with an infected person”	^11^	
LAIs	Two cases, 1981, German laboratory. Infected while processing stool samples	“From another, healthy family member an aerogenic, mannitol negative strain of *S. boydii* 14 (formerly Sachs A 12) was isolated which subsequently infected a laboratory technician when she was handling the fecal specimen. A second laboratory infection due to this organism occurred while performing the Serény test of pathogenicity. Both persons developed a severe dysenteric syndrome”	^21^	No
Chemical inactivation	Sonophotocatalytic disinfection	“…successfully developed a visible light assisted sonophotocatalysis (SPC) using Fe/ZnO nanoparticles (NPs) for the disinfection of *Shigella dysenteriae*. A consortia containing *S. dysenteriae* and *S. flexineri* was also completely disinfected using SPC. Growth conditions of *S. dysenteriae* like growth phases and growth temperature had different outcomes on the overall efficacy of SPC”	^23^	No
	Calcium hypochlorite at 70% active chlorine HTH	Solution at 0.05%: 7 g or 1/2 tablespoon in 10 L of water. Use for hands, skin clothes Solution at 0.2% 30 g or 2 tablespoons in 10 L of water use for floor, utensils, beds, personal belongings. Solution at 2%: 30 g or 2 tablespoons in 1 L water. Use for excreta, corpses	^6^	No
	Ozone treatment	“Treatments with ozone (1.6 and 2.2 ppm) for 1 min decreased *S. sonnei* population in water by 3.7 and 5.6 log cfu mL(-1), respectively… After 5.4 ppm ozone dose, lag-phases were longer for injured cells recovered at 10 degrees C than 37 degrees C. Furthermore, treated cells recovered in nutrient broth at 10 degrees C were unable to grow after 16.5 ppm ozone dose. Finally, after 5 min, *S. sonnei* counts were reduced by 0.9 and 1.4 log units in those shredded lettuce samples washed with 2 ppm of ozonated water with or without UV-C activation, respectively”	^24^	No
	Chlorinated lime at 30% active chlorine “bleaching powder”	Solution at 0.05%: 16 g or 1 tablespoon in 10 L of water. Use for hands, skin, clothes. Solution at 0.2%: 66 g or 4 tablespoons in 10 L of water. Use for floor, utensils, beds, personal belongings. Solution at 2%: 66 g or 4 tablespoons in 1 L of water. Use for excreta, corpses	^6^	No
	Sodium hypochlorite at 5% active chlorine “household bleach”	0.05%: 100 mL or 7 tablespoons in 10 L of water—Use for hands, skin, clothes 0.2%: 450 mL or 30 tablespoons in 10 L of water—Use for floor, utensils, beds, personal belongings 2%: 450 mL or 30 tablespoons in 1 L of water—Use for excreta, corpses	^6^	No
Thermal	Autoclave	Standard sterilization cycle of 121°C for 15 min, measured in the most difficult section of the load	^6^	Yes
	Steam	100°C for 1 h at normal atmospheric pressure	^22^	Yes

HTH, high test hypochlorite; LAIs, laboratory-acquired infections.

## Knowledge Gaps

### Decontamination and Inactivation

There appears to be a knowledge gap in the literature on the actual effectiveness, especially regarding concentrations and optimal contact times, of common disinfectants such as sodium hypochlorite, ethanol, glutaraldehyde, iodine, phenolics, and formaldehyde.

### Laboratory-Acquired Infection

The actual number of *Shigella* spp. LAIs are likely to be under-reported by staff since (1) shigellosis is generally self-limiting and not life threatening, (2) the origin of these infections is not often clear cut, and (3) it may cause embarrassment or fear of sanction. Although this is not strictly a gap, it limits the opportunities to determine the mechanisms and options to prevent LAIs.

## Conclusions

According to current guidance, it is recommended that a risk assessment be performed before beginning work with *Shigella* spp.^20^ Particular emphasis should be placed on PPE, handwashing, manipulation of faucet handles, and decontaminating work surfaces to decrease the risk of LAIs.^25^ For activities that may produce aerosols or infectious droplets, such as vortexing and pipetting of liquids containing *Shigella* spp., it is recommended that a biological safety cabinet be used.

Centrifugation operations should be performed using rotors with tight-fitting lids or safety cups in the case of bucket rotors.^25^ Since fewer reports of LAIs have been published formally or anecdotally (as per subject matter experts) most likely because biosafety guidance has relied more strongly on addressing specific risks and evidence collected in prior years, the recommendations described appear appropriate for handling organic materials containing *Shigella* spp.
